# Supplementary material for: Deep Learning-Based Tumor Segmentation of Murine Magnetic Resonance Images of Prostate Cancer Patient-Derived Xenografts
Source: Tomography. 2025 Feb 22;11(3):21. doi: 10.3390/tomography11030021 (PMC11946206; doi:10.3390/tomography11030021)
Supplement: Supplementary file 1 [file tomography-11-00021-s001.zip › tomography-3411234-supplementary.pdf]

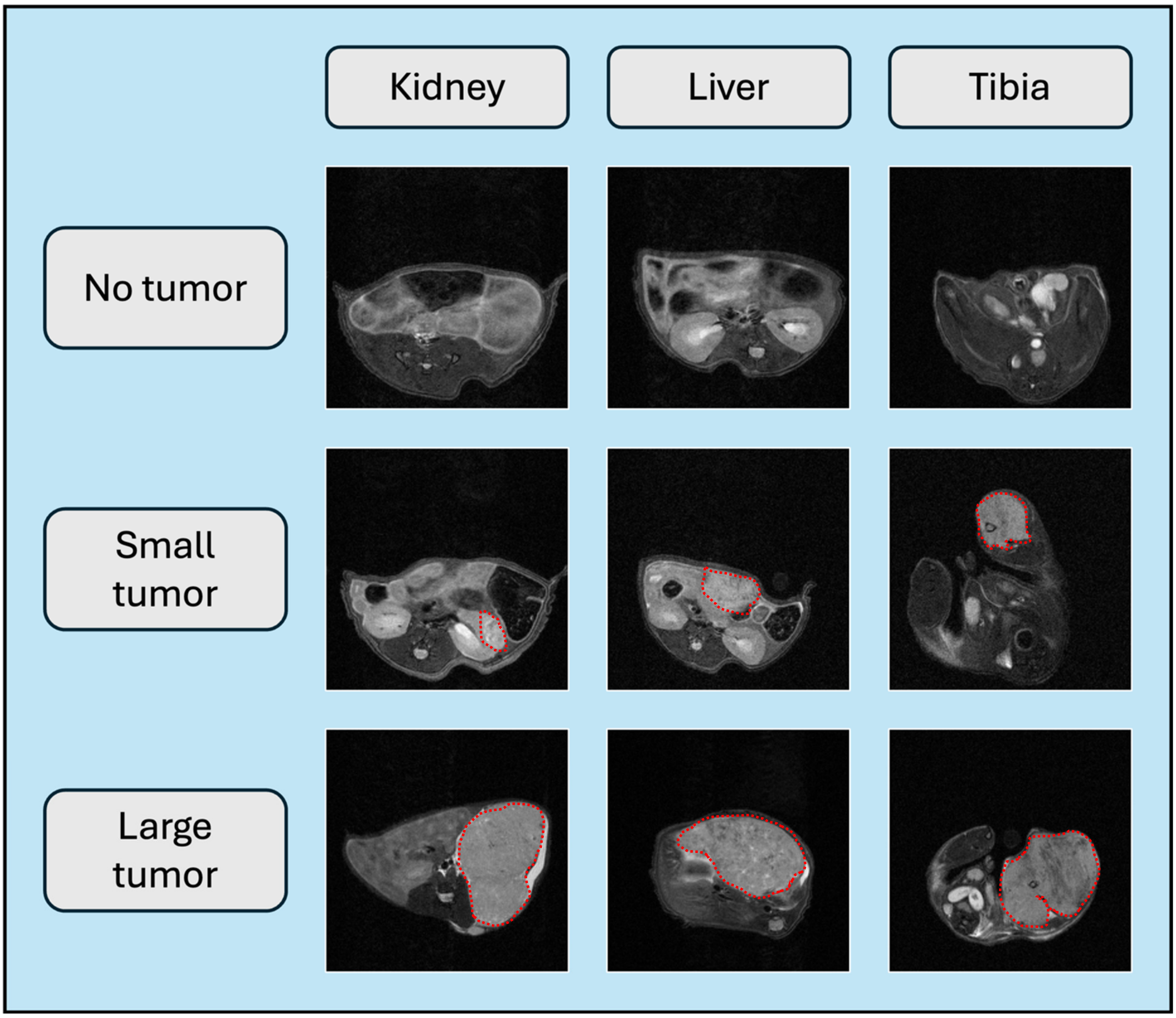

Figure S1. Representative images of individual MRI slices containing tumors of varying tumor volumes from each of the three inoculation sites. The red dotted line circumscribes the tumor.

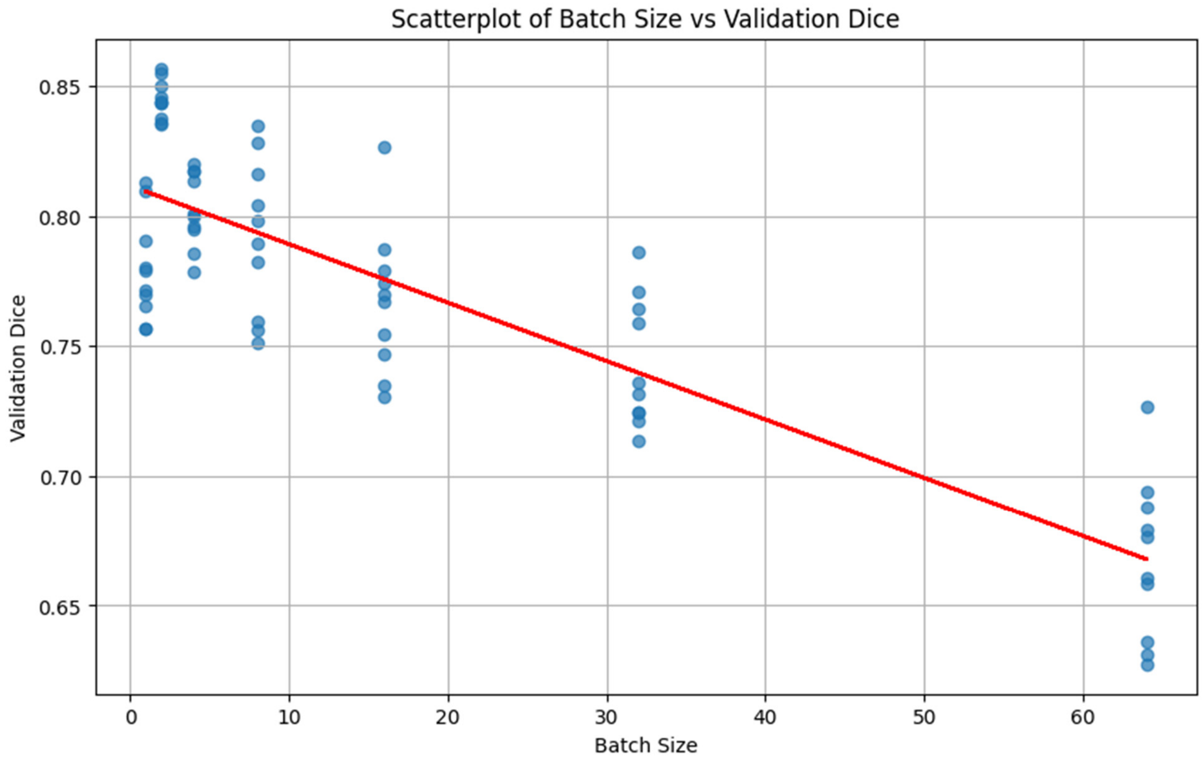

Figure S2. Scatter plot comparing effect of batch size on the validation dice score. Data was from experiments testing various optimizers and learning rates. The line of best fit, in red, shows a negative correlation between a larger batch size and the validation score, with a peak in the data occurring with a batch size of 2.

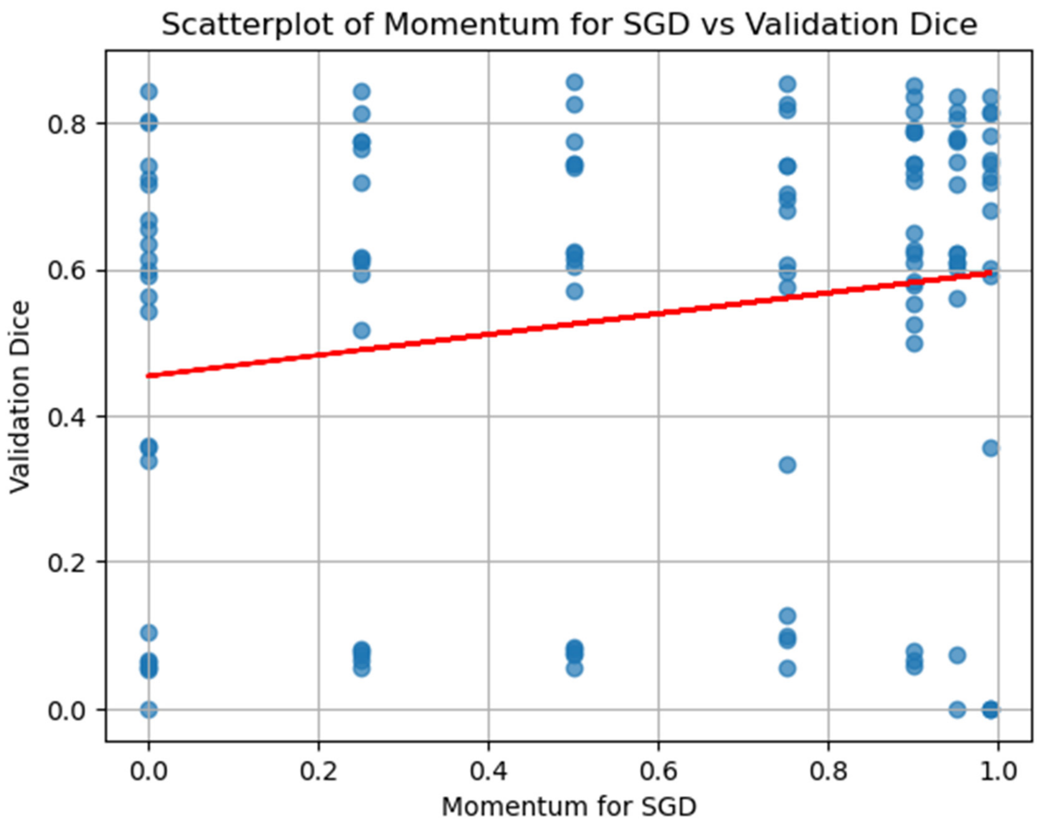

Figure S3. Scatter plot comparing the effect of different momentum values for the Schedule Gradient Descent optimizer (SGD) on the validation dice score. The line of best fit, in red shows a generally positive trend between a higher momentum value and the validation dice score.

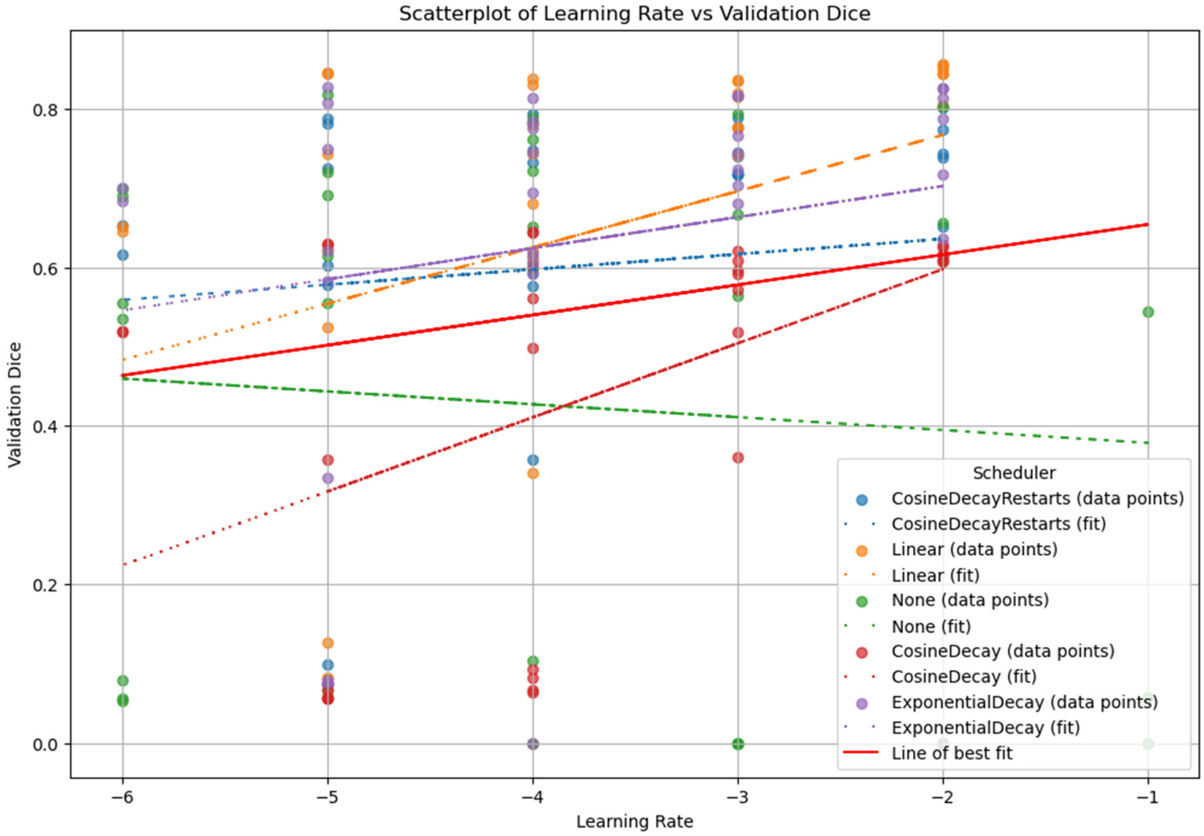

Figure S4. Scatter plot comparing the effect of the learning rate on the validation dice score, with different colors representing the choice of learning rate scheduler. The values displayed along the x-axis are the logarithm in base 10 of the learning rates we tested. The dotted lines represent the trend for each of the individual schedulers and the solid red line represents the overall trend across schedulers.

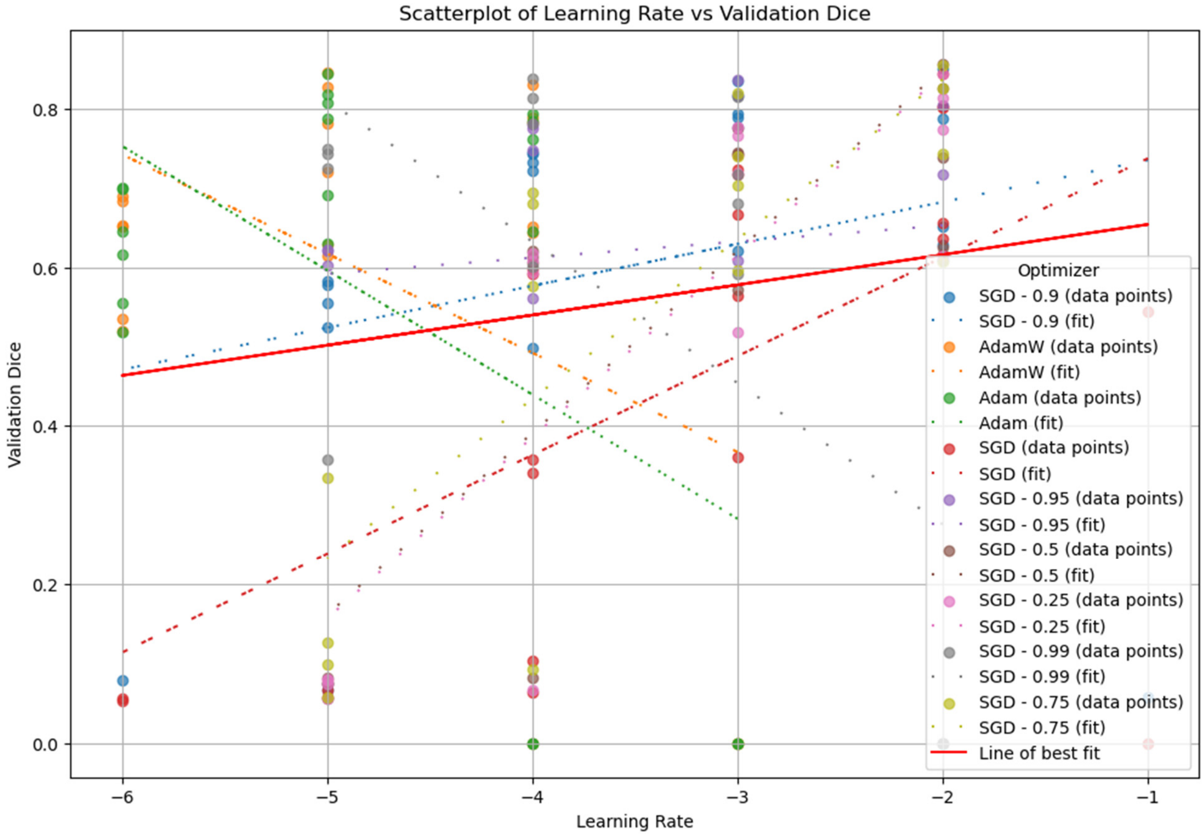

Figure S5. Scatter plot comparing the effect of the learning rate on the validation dice score, with different colors representing the choice of optimizer algorithm. The values displayed along the x-axis are the logarithm in base 10 of the learning rates we tested. The dotted lines represent the trend for each of the individual optimizers and the solid red line represents the overall trend across optimizers.
